# War Psychosis

**Published:** 1918-09

**Authors:** 


### NEUROLOGY AND PSYCHIATRY

**War Psychosis.** By Capt. D. K. HENDERSON. *Journal of Mental Science,* April, 1918, Vol. LXIV, N° 265.

Henderson has made a study of 202 cases of mental disorder occuring in Home Troops and his valuable results answer the pertinent question " Who shall be recruited? ".

His patients largely comprised those called up under Lord Derby's compulsory scheme and were confined to no one set of mental disorder, e. g., mental deficiency 61, dementia praecox 43, manic depressive 24, general paralysis 19, alcoholic insanity 77, traumatic insanity 12, psychoneuroses 10, paranoid states 8, toxic exhaustive insanity 3, epilepsy with insanity 3, organic brain disease 2.

These patients were all exposed practically to the same situation but each of them tended to react to that situation according to his inherent or predisposed constitution. He looks upon these cases not so much for their specific diagnosis or label as for " a reaction to a situation which could not be adequately met ".

In the *Mental deficiency* group the symptoms causing the patient to be sent to hospital were of an exceedingly transitory nature, 45 of 61 cases being sent home at the end of a few weeks.

The great majority of this group had very evident outward signs of gross abnormality. Special emphasis is laid on the tendency, after head injuries, for head symptoms to reassert themselves as soon as a patient is subjected to a strain that is too great for him to meet.

In discussing the dementia praecox group, Henderson rightly calls attention to the important fact that " their sudden, impulsive homocidal outbursts are of a particulary dangerous character. Many untoward accidents happening on active service and during training would never take place provided greater care were taken (in regard to those who were enlisted) and in apportioning to men work for which they are suited ".

Henderson suggests :

1. That cases showing mental deficiency, neuropathic and psychopathic traits, and giving a history and showing evidence of severe head injury, should for the most part be rigidly excluded from the army.

2. If it is necessary to recruit a certain number of these individuals, then it should be definitely ruled that under no circumstances should they be permitted to go on active service but should be given suitable work at home or at a base in France.

3. To effect the above object it is suggested that *a certain* number of mental specialists should be appointed to the recruiting boards, and recruiting medical officers generally should be given definite instructions to pay attention to all classes of probable mental defect or disorder and refer such cases to the expert.

4. At the large training camps there should be one mental specialist, whose business it would be to examine recruits and have those obviously unfit immediately rejected from the army.

5. It is only by adopting such methods as the above that we will ever come to grip with the wastage occurring in the army due to nervous and mental disease.

Note. These comments by Henderson are most pertinent and important, as it appears the R. A. M. C. have not adopted these methods. Every psychiatrist who has worked at the war-hospitals here could not help but be struck with the large number of obvious " dead weight " cases which have been unnecessarily, in fact criminally, dumped into the army.

